# Effectiveness of a primary care clinical ultrasound classroom for family physicians as a formative intervention system, a quasi-experimental trial: Study protocol

**DOI:** 10.1097/MD.0000000000019914

**Published:** 2020-07-02

**Authors:** Fernando Diego-Domínguez, Miguel Torrecilla-García, Jesús Casado-Huerga, Maria Ángeles Paule-Sánchez, Clara Isabel Soria-López, José Manuel Iglesias-Clemente, José María de Dios-Hernández, Natalia Diego-Mangas, María Cubillo-Jiménez, Fernando Pérez-Escanilla

**Affiliations:** a Instituto de Investigación Biomédica de Salamanca (IBSAL), Unidad de Investigación de Atención Primaria de Salamanca (APISAL); b Centro de Salud de San Juan de Salamanca; c Unidad Docente Multiprofesional de Atención Familiar y Comunitaria de Salamanca; d Centro de Salud de Periurbana Norte, Servicio de Salud de Castilla y León, Salamanca, Spain.

**Keywords:** family medicine, point-of-care ultrasound, primary care, training, ultrasonography

## Abstract

**Introduction:**

Clinical ultrasound is a technique that increases diagnostic capacity and facilitates clinical decision making. The objective is to develop and validate an ultrasound training methodology oriented to the clinical practice of the family physician.

**Methods:**

Quasi-experimental study, with a before/after design, a control group, and 1 year of follow-up. Twenty family physicians working in primary care health centers with a list of over 800 patients will be included, as well as a control group of family physicians with similar characteristics in terms of age, sex, and patient list. A structured training process oriented to the clinical practice of the family physician, primary care clinical ultrasound classroom (AECAP), will be carried out, and the improvement of knowledge and skills of the participants will be evaluated, as well as the improvement of the quality of care based on clinical indicators.

**Discussion:**

The family physician is in a privileged situation allows increasing the performance of ultrasound in frequent clinical situations and reducing care hours. We hope that the results obtained in this study demonstrate the effectiveness of the structured training method (AECAP) and support the generalization of ultrasound in primary health care.

**Ethics and dissemination:**

The study was approved by the Medical Research Ethics Committee of Salamanca on December 17, 2018 (cod 2018 11 134). The trial was registered in ClinicalTrials.gov provided by the US National Library of Medicine-number: NCT04283383.

## 1 Introduction

The use of ultrasound to aid the medical diagnosis poses an advancement in the diagnostic process, which facilitates and improves decision making in many areas of the usual clinical practice.^[1,2]^ The great possibilities of this technique, its innocuousness and its low cost, as well as its ease of use both in the doctor's office and at the patient's home, make it a first-line tool in the daily work of family physicians.^[2]^

The first publications that valued experiences and results of the use of ultrasound in primary care appeared in 1988 and refer to the training of doctors in obstetric ultrasound.^[3]^ From that point, a large number of studies have demonstrated that the use of ultrasound in the scope of primary care is a cost-effective diagnostic tool for multiple pathologies.^[4,5]^ Furthermore, its possibilities of use in family physician offices include a wide variety of clinical scenarios.^[6]^ Likewise, it has been demonstrated that, in some pathologies, whose paradigm could be cholelithiasis, the use of ultrasound by family physicians allows for an important reduction of care time.^[7]^ A specific use of ultrasound in family physician offices is the “point-of-care ultrasound” model (POCUS),^[8–10]^ which is characterized for focusing the technique on responding to a specific question, with brief examinations that guide ultrasound as a tool for clinical decision making and not only as a diagnostic test.^[11]^

Different studies show that, when this technique is performed by family physicians with enough training, the diagnostic findings for frequent pathologies are in line with those obtained by radiologists.^[12,13]^ Thus, there is a kappa index of over 0.8 for most pathologies,^[13,14]^ both in the diagnosis and in the follow-up of already diagnosed lesions.^[15]^ The worse levels of consistency have been reported in pancreatic and spleen pathology.^[13,16]^ Other studies have shown an increase of resolving capacity and a decrease of care time in certain processes and clinical scenarios, as well as a 50% decrease in referrals and applications for diagnostic tests.^[17,18]^

Therefore, multiple authors and scientific societies of primary care state that the generalized use of ultrasound in the usual practice of family physicians allows making clinical decisions with greater diagnostic precision and reducing the high levels of uncertainties with which these professionals work in the field of healthcare, as well as contributing to reducing the waiting lists of radiology services and shortening the care time in potentially critical situations.^[2,19,20]^

In the scope of primary health care in Spain, the use of ultrasound has been generalized in the healthcare centers of all the autonomous communities, which is also happening in other European countries.^[21]^ This is posing an important effort in the training of family physicians in the use of this tool. Currently, the ultrasound training offered by public and private entities for family physicians in Spain is abundant and of high quality, although it usually has a very academist orientation with little focus on the clinical practice.

The primary care clinical ultrasound classroom (PCCUC) presented by this project aims to provide the participating family physicians with specific training in ultrasound oriented to clinical practice in the scope of primary health care. The PCCUC uses a novel formative system characterized for the valuation and interpretation of clinical cases in real time (clinical session with the patient). Moreover, it includes a 1-year follow-up of the participants in order to assess the utility of this method not only in terms of the knowledge and skills acquired, but also regarding other results of clinical practice.

Therefore, the main objective of the project is to develop an ultrasound training methodology oriented to the clinical practice of family physicians in the scope of primary health care; it also aims to evaluate the effectiveness of such training regarding the increase of knowledge and skills and the improvement of the clinical practice, evaluated by the number of referrals to specialized radiology services, diagnostic waiting time, diagnostic precision, and improvement of the health care provided.

As a secondary objective, the project aims to validate the PCCUC training system for use in the training of undergraduates and post-graduates and in the continuous training of family physicians.

## 2 Methods

### 2.1 Design and scope

This is a quasi-experimental study with a before/after design, a group control and a 1-year follow-up to evaluate the effectiveness of the formative intervention in clinical ultrasound with family physicians. The control group will consist of family physicians with similar characteristics, who will not participate in the AECAP. The project will be carried out with family physicians who work in Spanish primary healthcare centers.

### 2.2 Study population

The study will include 20 family physicians who want to participate voluntarily, meeting the following inclusion criteria: the practitioners must work in a Spanish primary healthcare center where ultrasonography is available and which belongs to the Health Service of Castilla y León (Sacyl) and to the Health Area of Salamanca; the participants must have a list of over 800 patients; they must have no previous training in clinical ultrasound; and they will have to sign a commitment of attendance and participation in the PCCUC. A control group will be recruited, which will be composed of physicians with similar characteristics in terms of age, sex, workplace, and number of patients assigned. They will be recruited in the first quarter of 2020.

### 2.3 Intervention

The intervention designed in the AECAP consists of a basic structure and a specific methodology, which will be carried out in the classroom of the San Juan Healthcare Centre in Salamanca.

#### 2.3.1 Structure

The material required to perform the formative activity will be an Esaote MyLabSix ultrasound scanner (Esaote Spain, Barcelona, Spain), a Esaote MyLabGamma portable ultrasound scanner, a 56-in. high-resolution TV HDMI connection to the ultrasound scanner, a computer connected to the ultrasound scanners and to the TV, and an examination table.

#### 2.3.2 Formative methodology of the AECAP

The ultrasound classroom will be attended twice per month, specifically on the second and fourth Tuesday of each month, from 6 p.m. to 8.30 p.m. At the beginning of each AECAP, the participants will be given the necessary material to complete the ultrasound training individually, following the structure described below.

Basic course in clinical ultrasound (abdomen and thyroid ultrasonography) of a total duration of 30 hours (15 hours of theory and 15 hours of practice). The course will be taught in the first term of 2020.

##### 2.3.2.1 Clinical sessions with patients

**Figure 1:**
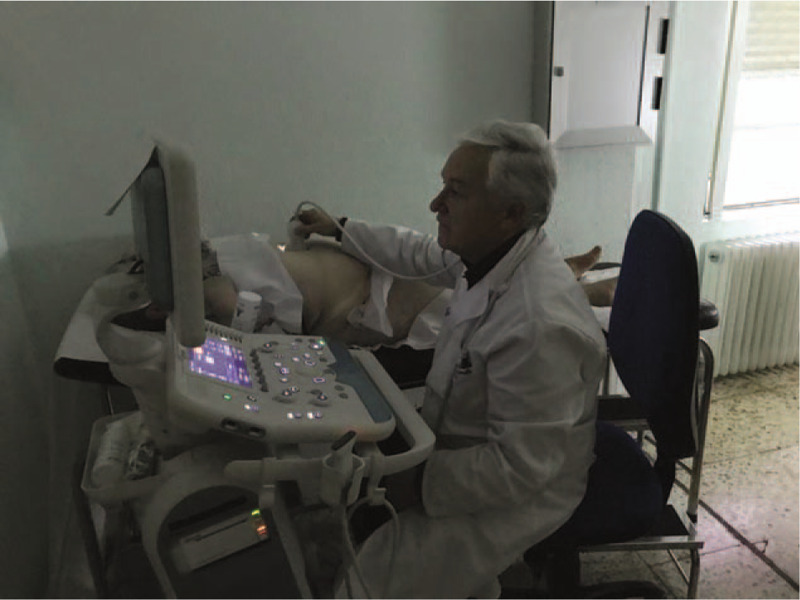
Clinical ultrasound training in the primary care clinical ultrasound classroom (AECAP).

In this stage, the participants will familiarize with the practical utility of clinical ultrasound through clinical cases with real patients, who agreed to participate by signing an informed consent document. The sessions will be presented as follows: before the patient enters the classroom, the clinical case is presented to the participating physicians, the patient enters the classroom, the ultrasound is carried out with visualization in the TV of the classroom, and the result is written in a report (Fig. 1). All the data will be incorporated to the “case presentation model.” This phase will be conducted from April to October 2020. The teacher of each session will collect the informed consent of the participating patients.

##### 2.3.2.2 Final practical lecture

During the formative process, all participants will have to present a clinical case in which the use of ultrasound is useful for the diagnosis, and which will be used to assess the formative process. In each clinical case, the following variables will be recorded: relevance of ultrasound in the case, consistency of the results with the clinical data and therapeutic plan, and the need for ultrasound to be carried out by the radiology service or application for other imagery tests (CT, NMR) and endoscopy.

##### 2.3.2.3 Evaluation of the intervention

Primary and secondary results.

###### 2.3.2.3.1 Primary results

Evaluation of knowledge and skills. An initial evaluation of knowledge will be carried out prior to the beginning of the formative intervention, which will consist of 10 multiple-choice questions with only 1 possible answer (5 theoretical questions about the fundamentals and use of ultrasonography, and interpretation of 5 images of pathologies or ultrasound anatomy). Once the formative sessions and practical lectures begin, a continuous evaluation system will be used, through which the participants will be evaluated by the teachers/instructors, valuing, in a normalized manner, theoretical, practical, and interpretative aspects. For the final evaluation, a new test will be carried out, with 10 questions similar to those of the initial test (5 theoretical questions and 5 common images). Then, a structured valuation will be carried out for the presentation of the final clinical case of each participant. Lastly, the ultrasound examination of 6 patients with common pathologies or normal ultrasound, who have an abdominal or thyroidal ultrasound previously informed by the radiology service, will be evaluated, assessing the consistency of the description, measures, and diagnosis.

###### 2.3.2.3.2 Secondary results

Evaluation of results in the clinical practice.

-Number of applications to the radiodiagnosis service: the number of applications of abdominal and thyroidal ultrasound scans sent to the radiology service by the physicians in the PCCUC and those in the control group in the year before and the year after the training process will be studied.
-Number of referrals to specialized health care: In the same annual periods, the number of referrals by both groups of family physicians will be valued.
-Diagnosis waiting times of physicians of the intervention and control groups for 2 processes, which will be used as indicators of healthcare quality improvement (cholelithiasis and the etiological diagnosis of haematuria). The source of data will be the computerized clinical history of the Health Service of Castilla y León.

### 2.4 Data analysis

The results will be expressed as mean ± standard deviation for normally distributed quantitative variables or medians (inter-quartile ranges) for biased variables and by distribution of frequency for qualitative variables. For the comparison of qualitative variables, we will use the Chi-squared test or Fisher exact test whenever necessary. To analyze the consistency of the diagnoses, we will use the Kappa test. In the comparison of qualitative variables, we will use the Student *t* test or the Mann–Whitney's *U* test when the conditions of normality of the variable are not met. For the analysis of repeated quantitative before/after data, we will use the Student *t* of paired data or the Wilcoxon test whenever necessary, and the McNemar test for qualitative data. The analysis of the correlation between quantitative variables will be carried out using the Pearson correlation and the Spearman test when the conditions of normality are not met. Lastly, a multivariate analysis will be conducted to determine the factors that produce the best result in the formative process.

The statistical analysis will be carried out using the SPSS v.23.0 software (IBM Corp., Armonk, NY) with the level of statistical significance established at *P* < .05.

### 2.5 Ethics and dissemination

The study was approved by the Medical Research Ethics Committee of Salamanca on December 2018. A SPIRIT checklist is available for this protocol. The trial has been registered in ClinicalTrials.gov with identifier NCT04283383. The participants must sign an informed consent document prior to their inclusion in the study, in accordance with the Declaration of Helsinki. The subjects will be informed about the objectives of the study and the risks and benefits of the examinations that will be carried out, including sample collection. None of the tests could result in mortal damage to the subjects. The confidentiality of the subjects will be guaranteed at all times in compliance with the current laws and regulations on personal data protection (LOPD 3/2018 of December 5, 1999) and the conditions described in Law 14/2007 of biomedical research.

## 3 Discussion

As they provide longitudinal and integral health care to their patients, family physicians are in a privileged situation that allows increasing the performance of ultrasonography^[2]^ in frequent clinical situations and reducing the care time.^[7]^

Groups of experts are making an important effort to define the clinical scenarios in which ultrasonography is most effective in the scope of primary health care,^[6,11]^ approaching this technique as a tool that practitioners use in the diagnostic process of POCUS.^[10,11]^ In this sense, some autonomous communities of Spain, such as Castilla y León, already include clinical ultrasonography in their list of primary healthcare services (Table 1).^[22]^

**Table 1:**
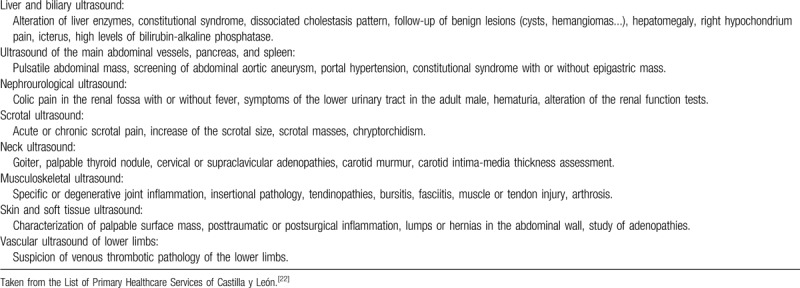
Main indications of clinical ultrasound in primary health care.

Therefore, the training of family physicians in clinical ultrasound is a necessary condition for the effective implementation of this tool in primary healthcare centers.^[2]^ Similarly, the new portable devices that, as the future stethoscopes, allow conducting high-quality ultrasound scans using an ultrasound scanner coupled to a Smartphone,^[23,24]^ seem to be designed for use by family physicians and create new possibilities of clinical use both in the doctor's office and at the patient's home.

In the field of primary health care, there are very few studies that value the results of a formative process in clinical ultrasound.^[13]^ This study, as was previously commented, aims to validate a clinical ultrasonography training system for primary healthcare practitioners (AECAP), based on practices with real patients and clinical session methodology; moreover, it is intended to measure certain indicators of consistency, efficiency, and resolving capacity, with a before/after-intervention system using a control group to reinforce the results. We expect that the results obtained in this study will demonstrate the efficacy of the structured training method (PCCUC) and provide arguments in favor of generalizing ultrasonography as a tool of daily use in primary health care.

### 3.1 Methodological limitations

Since this is a quasi-experimental study with a great variability of the clinical practice of family physicians, it cannot be guaranteed that the differences obtained will be due exclusively to the formative intervention. Likewise, we cannot discard the possibility that a certain degree of Hawthorne effect may occur among the participants. Lastly, the fact that it is impossible to normalize the continuous evaluation at 100% poses a limitation to the objectivity of this result.

### 3.2 Dissemination plan

We will use a variety of methods to guarantee that our work achieves maximum visibility. The publication of the study protocol provides an important first step in this sense. Through this document, we aim to show a general view of the relevant literature, and, at the same time, highlight the current research needs that required the design and implementation of this study.

The results of the study, given their applicability and implications for the development of primary health care, as well as for the general population, will be disseminated in gatherings and meetings of researchers and in articles published in peer-reviewed scientific journals. We intend to publish at least 1 manuscript with the main findings and 1 manuscript with the secondary findings. The authors of the protocol will be responsible for the publication of the paper for the dissemination of the results of the study.

## Acknowledgments

The authors thank all the professionals and patients who participate in this study.

## Author contributions

Fernando Diego-Dominguez, Miguel Torrecilla-García, Jesús Casado-Huerga, and Fernando Perez Escanilla, contributed to the conception or design of the work. Fernando Diego-Dominguez, Miguel Torrecilla-García, Jesús Casado-Huerga, Maria Ángeles Paule-Sánchez, Clara Isabel Soria-López, José Manuel Iglesias-Clemente, José María de Dios Hernández, Natalia Diego-Mangas, María Cubillo-Jimenez, and Fernando Perez Escanilla contributed to the acquisition, analysis, or interpretation of data for the work. Fernando Diego-Dominguez and Fernando Perez Escanilla drafted the manuscript. Miguel Torrecilla-García, Jesús Casado-Huerga, Clara Isabel Soria-López, José Manuel Iglesias-Clemente, José María de Dios Hernández, critically revised the manuscript. All authors gave final approval and agree to be accountable for all aspects of the work ensuring integrity and accuracy.
